# Predictors of long-term decannulation in patients with disorders of consciousness

**DOI:** 10.3389/fneur.2023.1099307

**Published:** 2023-10-02

**Authors:** Ying Chen, Gulijiakela Aishan, Shunjuan Fan, Tingwei Wang, Junfa Wu, Chinhsuan Chia, Gang Liu, Li Wang, Ruiping Hu

**Affiliations:** Department of Rehabilitation Medicine, Huashan Hospital, Fudan University, Shanghai, China

**Keywords:** disorders of consciousness, tracheostomy, decannulation, predictors, dysphagia

## Abstract

**Purpose:**

The predictors of tracheostomy decannulation in patients with disorders of consciousness (DOC) are not comprehensively understood, making prognosis difficult. The primary objective of this study was to identify predictors of tracheostomy decannulation in patients with disorders of consciousness (DOC). The secondary aim was to evaluate the feasibility and safety of the modified Evans blue dye test (MEBDT) in tracheostomized DOC patients.

**Methods:**

This retrospective study included all patients with disorders of consciousness (DOC) who underwent tracheostomy and were admitted between January 2016 and September 2022. Age, sex, etiology, initial Glasgow coma scale (GCS), initial Coma Recovery Scale-Revised (CRS-R), diagnosis of unresponsive wakefulness syndrome (UWS) or minimal consciousness state (MCS), MEBDT, initial modified Rankin scale (mRS), and initial Functional Oral Intake Scale (FOIS) were collected upon study enrollment. The relationship between clinical characteristics and cannulation status was investigated through a Cox regression model.

**Results:**

A total of 141 patients were included in the study. The average age of these patients was 52.5 ± 16.7 years, with 42 (29.8%) being women. During the study period, 86 subjects (61%) underwent successful decannulation. Univariate analysis revealed that decannulated patients exhibited a significantly better conscious state compared to those without decannulation (CRS-R: *p* < 0.001; GCS: *p* = 0.023; MCS vs. UWS: *p* < 0.001). Additionally, a negative modified Evans blue dye test (MEBDT) result was significantly associated with tracheostomy decannulation (*p* < 0.001). In the multivariate analysis, successful decannulation was associated with a higher level of consciousness (MCS vs. UWS, *p* < 0.001, HR = 6.694) and a negative MEBDT result (negative vs. positive, *p* = 0.006, HR = 1.873). The Kaplan–Meier analysis further demonstrated that MEBDT-negative patients and those in the MCS category had a higher probability of decannulation at 12 months (*p* < 0.001).

**Conclusion:**

The findings of this study indicate that a negative MEBDT result and a higher level of consciousness can serve as predictive factors for successful tracheostomy decannulation in DOC patients.

## Background

A disorder of consciousness (DOC) refers to a state of altered consciousness resulting from neural system dysfunction or injury that affects arousal and awareness (1). Following the acute coma phase, patients may transition to a prolonged DOC state, encompassing the unresponsive wakefulness state (UWS) and the minimally conscious state (MCS). Tracheostomy is commonly performed in patients with DOC (2), and it has been associated with improved survival rates following severe brain injury (3). However, long-term tracheostomy carries an elevated risk of complications, including the formation of granulation tissue, tracheal stenosis, tracheomalacia, tracheoesophageal fistula, and aspiration (4). Additionally, it is associated with prolonged hospital stays (5), increased infection rates (6), and escalating healthcare costs (7). The presence of tracheostomy may adversely affect the rehabilitation process (8, 9). Therefore, decannulation is a crucial objective during the post-acute stages of rehabilitation (10). Predictors for decannulation include effective cough (8, 11, 12), absence of severe dysphagia (8, 11, 12), Glasgow coma scale (GCS) ≥ 8 (8, 11), higher conscious state according to the Coma Recovery Scale-Revised (CRS-R) (13), younger age (11), and etiology of traumatic brain injury (11). Nevertheless, researchers lack consensus on the predictors for tracheostomy decannulation in patients with DOC (14). The early identification of DOC patients eligible for decannulation is an area that has been largely unexplored until now. To address this research gap, we conducted a study on a substantial cohort of patients with DOC to determine the factors that can predict safe decannulation.

The modified Evans blue dye test (MEBDT) has emerged as a standard clinical tool for evaluating patients with tracheostomy and suspected dysphagia due to its economic, simple, and readily available nature (15). However, the literature provides limited reports on the feasibility and safety of using MEBDT in DOC patients. Thus, a second objective of this study was to assess the feasibility and potential adverse effects of a novel MEBDT method in tracheostomized patients with DOC resulting from diverse etiologies.

## Materials and methods

### Study design

This study used a retrospective review of tracheostomized patients with disorders of consciousness, adhering to the STROBE (Strengthening the Reporting of Observational Studies in Epidemiology) guidelines (16). The study received approval from the Ethics Committee of Huashan Hospital, affiliated with Fudan University, and written informed consent was obtained from all legal surrogates.

### Patients

The patients were consecutively recruited from January 2016 to September 2022 at the Neurorehabilitation Department of Huashan Hospital in Shanghai, China. Inclusion criteria consisted of a confirmed diagnosis of prolonged disorders of consciousness (DOC) (17) (defined as at least 28 days post-brain injury) using CRS-R, presence of tracheostomy cannula, age of 18 years or older, absence of invasive ventilation, clinical stability (18) (defined as cardio-circulatory stability, absence of organ failure, absence of septic failure, SpO_2_ > 90%), and at least 12-month follow-up.

### Decannulation protocol

The decannulation protocol employed in this study was based on previous literature (19). The protocol consisted of two steps. The first step involved deflating the cuff of the tracheostomy tube. If there was no active respiratory infection present, the protocol proceeded to the second step, which involved replacing the cuffed tracheal tube with an uncuffed one. The second step of the protocol was the capping test, during which the duration of capping was gradually extended for most DOC patients. Throughout the capping test, regular assessments including blood and culture tests, blood gas analysis, and chest radiography were conducted to identify and detect pneumonia. In cases where patients showed intolerance to capping, flexible fiberoptic bronchoscopy was utilized to assess the airway. Decannulation was performed after a minimum of 48 consecutive h of capping without experiencing desaturation or stridor. Prolonged tracheostomy in our patients can commonly occur due to recurrent aspiration pneumonia, a poor level of consciousness, or intolerance to the capping test.

### Data collection

Baseline data on admission included information on age, sex, etiology, GCS, CRS-R, and MEBDT results. To differentiate between patients in a state of UWS or MCS, repeated examinations of the CRS-R were conducted. In our study, five assessments were performed over a span of several days (5 to 10 days). The CRS-R scale was administered by a specialized doctor during hospitalization and follow-up. An assessment of liquid aspiration was conducted using the modified Evans blue dye test. This test was performed by a nurse and an experienced speech-language pathologist (SLP). Initially, patients were positioned with the head of the bed elevated at least 30°. Unless otherwise specified, the tracheostomy cuff was deflated. Then, 1 mL of thin liquid with 2 drops of blue food coloring was placed in a 1 mL syringe without a needle. The syringe was positioned at the posterior part of the patient’s tongue. Immediately after administration, tracheal suctioning was performed, followed by additional suctioning at 30 and 60 min thereafter. Tracheal secretions were continuously monitored for the presence of the blue dye for 24 h. The presence of blue dye in any tracheal secretions indicated a positive result for the MEBDT (20).

At the 12-month follow-up visit, tracheostomy decannulation, or decannulation failure, decannulation time was recorded. The primary outcome of interest was tracheostomy decannulation, which was defined as the absence of a tracheostomy at the last follow-up visit.

### Statistical analysis

Baseline clinical characteristics were compared between patients with successful decannulation after tracheostomy and patients without decannulation over a 12-month follow-up period. All statistical analyses were conducted using SPSS software, version 17.0. Group comparisons were presented as mean (standard deviation) and analyzed using Student’s *t*-test, or as median (interquartile range) and analyzed using the Mann–Whitney U-test, based on the Shapiro–Wilk test of normality. The successful decannulation was defined as the outcome; cox regression analysis and Kaplan–Meier analysis was used to evaluate DOC patient predictors on the outcome of interest: tracheostomy decannulation. Variables that demonstrated statistical significance (*p* < 0.05) in the univariate analysis were included in the multivariate analysis using Cox regression. A significance level of a *p*-value of <0.05 was set for all statistical tests.

## Results

A total of 149 DOC patients were consecutively recruited in this study; eight of them were lost at the 12-month follow-up after discharge. The remaining 141 patients were included in the analysis. The average age of the patients was 52.5 ± 16.7 years, and 42 (29.8%) of them were women. The median CRS-R score was 9, with 48.2% of patients having a GCS score of 8 or higher. The median mRS score was 5, and the median FOIS score was 1 at admission. The distribution of diagnoses among the patients was as follows: stroke in 76 cases (53.9%), traumatic brain injury (TBI) in 42 cases (29.8%), hypoxic–ischemic encephalopathy (HIE) in 12 cases (8.5%), and other etiologies in 11 cases (7.8%). Based on repetitive assessments of the CRS-R, the patients were initially classified as UWS in 20.6% of cases and MCS in 79.4% of cases. Among the patients, 73.8% tested positive in the modified Evans blue dye test. Table 1 provides a summary of the clinical and demographic characteristics of the study population.

**Table 1:** Patient characteristics.

Characteristic/subgroup	Total (*n* = 141)	Decannulation (*n* = 86)	Non-decannulation (*n* = 55)	*p*-value
Age, years (x ± s)	52.5 ± 16.7	49.4 ± 17.0	57.3 ± 15.0	0.156
Sex (F/M)	42/99	26/60	16/39	0.520
Time to decannulation, days	/	132 (76;204.7)	/	/
Median (P25;P75)				
MEBDT result				<0.001
Positive [*n* (%)]	104 (73.8%)	54 (62.8%)	50 (90.9%)	
Negative [*n* (%)]	37 (26.2%)	32 (37.2%)	5 (9.1%)	
CRS-R, median (P25;P75)	9 (8;10)	9 (9;10)	8 (5.5;9)	<0.001
UWS [*n* (%)]	29 (20.6%)	4 (4.7%)	25 (45.5%)	<0.001
MCS [*n* (%)]	112 (79.4%)	82 (95.3%)	30 (54.5%)	<0.001
GCS				0.023
GCS ≥ 8 [*n* (%)]	68 (48.2%)	49 (56.9%)	20 (36.4%)	
GCS < 8 [*n* (%)]	73 (51.8%)	37 (43.1%)	35 (63.6%)	
mRS	5 (5;5)	5 (5;5)	5 (5;5)	1.0
Median (P25;P75)				
FOIS	1 (1;1)	1 (1;1)	1 (1;1)	0.256
Median (P25;P75)				
Etiology [*n* (%)]				0.425
Stroke [*n* (%)]	76(53.9%)	44 (51.1%)	32 (58.2%)	
TBI [*n* (%)]	42 (29.8%)	28 (32.6%)	14 (25.4%)	
HIE [*n* (%)]	11 (7.8%)	5 (5.8%)	6 (10.9%)	
Others [*n* (%)]	12 (8.5%)	9 (10.5%)	3 (5.5%)	
Mortality [*n* (%)]	3 (2.1%)	/	3 (5.5%)	0.57
Complications [*n* (%)]	3 (2.1%)	1(0.7%)	2 (1.4%)	0.456

MEBDT, modified Evans blue dye test; CRS-R, Coma Recovery Scale-Revised; UWS, unresponsive wakefulness syndrome; MCS, minimally conscious state; GCS, Glasgow Coma Scale; mRS, modified Rankin scale; FOIS, Functional Oral Intake Scale; TBI, traumatic brain injury; HIE, hypoxic–ischemic encephalopathy.

During and after the modified blue dye test, a total of three patients (2.1%) experienced mild adverse effects. Each patient reported a single mild adverse effect, which included blood-tinged sputum, nausea, and dyspepsia. However, no patients experienced any major adverse effects such as fever or aspiration pneumonia following the MEBDT tests, as confirmed by radiological examinations including pulmonary CT or chest X-ray. The overall mortality rate in the study was 2.1%, with three patients experiencing adverse outcomes. One patient was diagnosed with cardiopulmonary arrest, while the other two patients were diagnosed with multiple organ failure.

### Group comparison: decannulation versus non-decannulation

**Figure 1:**
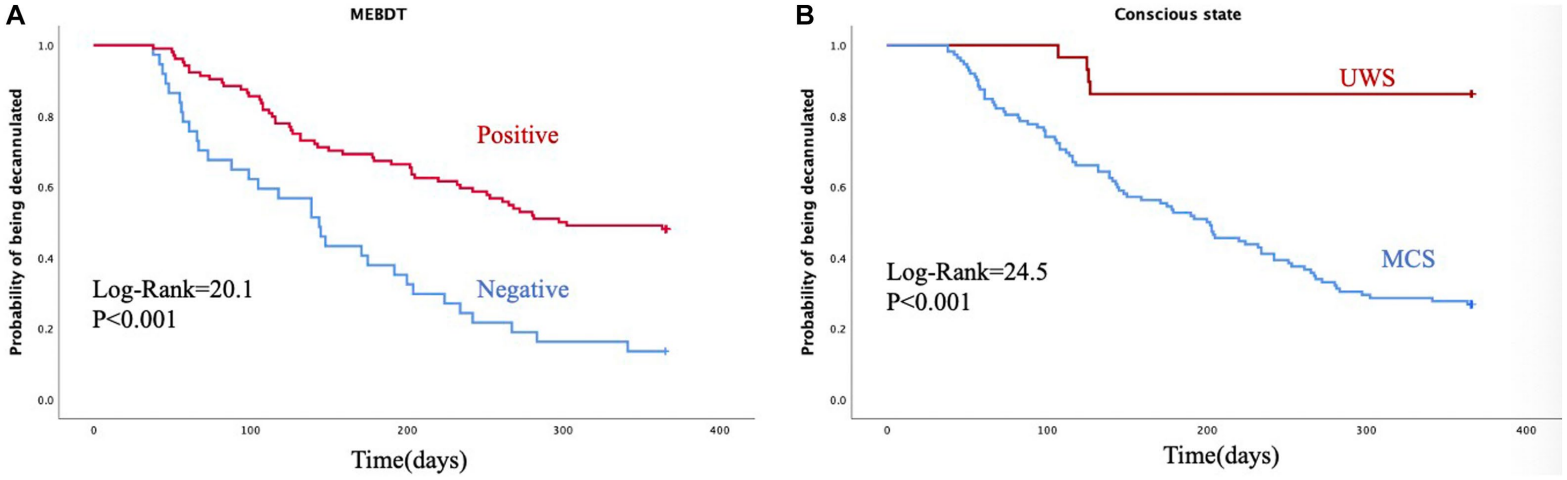
Kaplan-Meier analysis of the probability of decannulation in the two groups. **(A)** shows a higher probability of occurrence of decannulation in the MEBDT-group.**(B)** shows a higher probability of occurrence of decannulation in the MCS group.

In the 12-month follow-up period, decannulation was successfully performed on 86 subjects (D), accounting for 61% of the participants. However, in 55 cases, decannulation was not possible (ND), making up 39% of the participants. One decannulated patient required emergency re-cannulation due to respiratory failure. There were no significant differences observed in terms of sex, age, etiologies, mortality, initial mRS score, initial FOIS score, and complications associated with the modified Evans blue dye test between the D and ND patient groups. However, decannulated patients exhibited a higher level of consciousness compared to patients without decannulation, as indicated by the CRS-R score (*p* < 0.001) and the GCS score (*p* = 0.023). Additionally, a significantly higher proportion of decannulated patients were in a minimally conscious state (MCS) compared to the group of patients in a vegetative state (UWS) (95.3% vs. 54.5%, *p* < 0.001). The proportion of negative MEBDT results was significantly greater in the D group than in the ND group (37.2% vs. 9.1%, p < 0.001). The D group had a median time to decannulation of 132 days (interquartile range: 76 to 204.7 days). Kaplan–Meier analysis (Figure 1A) demonstrated a higher probability of decannulation at 12 months for patients with negative MEBDT results compared to those with positive results (*p* < 0.001). Furthermore, the probability of decannulation was higher in MCS patients compared to UWS patients (*p* < 0.001) (Figure 1B).

### Cox regression analysis

Variables including sex (female vs. male), age (<50 vs. ≥50 years), GCS score (GCS ≥ 8 vs. GCS < 8), etiology (others vs. TBI), conscious state (MCS vs. UWS), and modified Evans blue dye test results (negative vs. positive) were included as variables in the univariate Cox regression analysis (see Table 2).

**Table 2:** Univariate Cox regression analysis.

Characteristics	HR(95% CI)	*p*-value
Sex (male vs. female)	1.053 (0.664–1.668)	0.827
Age (<50 vs. ≥50 years)	1.432 (0.877–2.055)	0.175
GCS score(GCS ≥ 8 vs. GCS < 8)	0.405 (0.186–0.878)	0.022
Etiology (TBI vs. others)	0.907 (0.577–1.424)	0.670
MEBDT(negative vs. positive)	2.646 (1.700–4.118)	<0.001
Consciousness(MCS vs. UWS)	8.437 (3.085–23.072)	<0.001

Univariate Cox regression analysis of the patient predictors on the outcome of interest: tracheostomy decannulation. HR, hazard ratio; TBI, traumatic brain injury; GCS, Glasgow Coma Scale; MEBDT, modified Evans blue dye test; MCS, minimally conscious state; UWS, unresponsive wakefulness syndrome.

In the multivariate Cox regression analysis, the conscious state (MCS vs. UWS), GCS score (GCS ≥ 8 vs. GCS < 8), and MEBDT results (negative vs. positive) were included as variables. The analysis revealed that both the conscious state and MEBDT results had a statistically significant relationship with decannulation (*p* < 0.001, HR = 6.694 and *p* = 0.006, HR = 1.873, respectively). However, the GCS score only showed a weak association with decannulation (*p* = 0.115). These findings are summarized in Table 3.

**Table 3:** Multivariate Cox regression analysis.

Predictors	HR (95%CI)	*p*-value
MEBDT (negative vs. positive)	1.873 (1.197–2.928)	0.006
Consciousness (MCS vs. UWS)	6.694 (2.408–18.605)	<0.001
GCS score (GCS ≥ 8 vs. GCS < 8)	0.536 (0.246–1.165)	0.115

Patient characteristics that were significant in Table 2 were included in multivariate Cox regression analysis. The hazard ratio is for decannulation.

## Discussion

The current literature lacks comprehensive information regarding decannulation in patients with prolonged tracheostomy (21). There is no standardized set of clinical parameters to predict the success of decannulation in patients with disorders of consciousness. The conscious state (8) and the improvement of DOC (14, 22) have been identified as crucial factors. However, some studies have reported successful decannulation in patients with a state of UWS (23–25). Interestingly, in these cases, decannulated UWS patients had lower mortality rates compared to those who remained tracheostomized (14). In our study, we observed successful decannulation in four UWS patients out of a total of 29 patients (4.7%). However, the majority of UWS patients did not undergo decannulation during the 12-month follow-up period. In our sample, no significant difference was found in the mortality rate between UWS patients who underwent tracheostomy decannulation and patients with UWS who remained tracheostomized. Two assessment scales for measuring conscious state were used in our study: GCS and CRS-R. The univariate analysis results indicated a strong association between assessments of consciousness (including GCS and CRS-R) and tracheostomy decannulation. However, in multivariate analysis, the conscious state (MCS vs. UWS) according to repetitive CRS-R was found to have a statistically significant relationship with decannulation, while the GCS (GCS ≤ 8 vs. GCS > 8) showed a weak association. This result is likely because the Coma Recovery Scale-Revised (CRS-R) is more sensitive in detecting signs of consciousness compared to the GCS (17). Some authors (18, 26) have reported no significant differences in GCS between the decannulated (D) and non-decannulated (ND) groups. However, there are divergent findings from other authors. They have suggested that a higher GCS is associated with a higher likelihood of decannulation (8, 12, 19, 27). In contrast to GCS, which has shown inconsistent results in different literature, CRS-R has demonstrated a high degree of consistency in predicting decannulation across the literature (13, 19). Therefore, CRS-R appears to be a better predictor of decannulation than GCS for patients with disorders of consciousness.

The previous study (8) suggested that the blue dye test (BDT) was a reliable predictor for decannulation in patients with acquired brain injury (ABI). In our study, a strong association between negative MEBDT results and decannulation was also demonstrated. Furthermore, in the multivariate analysis, the MEBDT results (negative vs. positive) showed a statistically significant relationship with decannulation. Therefore, based on these findings, a negative MEBDT result could be considered as a predictor for decannulation in DOC patients.

One of the objectives of this study was to assess the feasibility and safety of MEBDT in DOC patients. While the use of the blue dye test (BDT) is common in tracheostomized patients with various conditions such as pulmonary disease (28, 29), cardiovascular disease, and nervous system diseases (29, 30), limited research has specifically focused on its application in DOC patients. Previous studies, such as Enrichi’s study (8), employed the original Evans blue dye test method in patients with ABI, while others, such as Hakiki (19), did not specify the method of BDT used. In our clinical practice, we initially utilized Belafsky’s (31) method which involved administering 45 mL of ice chips to three DOC patients. However, we encountered difficulties with swallowing in these individuals. Consequently, we developed a modified approach using 1 mL of thin liquid with two drops of blue food coloring. Both patients in a vegetative state and those in a minimally conscious state exhibited dysfunction in the oral phase of swallowing (32). Recognizing an effective oral phase as a potential indicator of consciousness in DOC diagnosis (33), our modified MEBDT method facilitated swallowing in individuals with impaired consciousness. Importantly, our study demonstrated that the MEBDT was easy and safe to administer in unconscious subjects, thus providing valuable insights into its feasibility in the DOC population.

Retrospective, observational studies have limitations as they rely on data collected in the past and may not have controlled all possible confounding factors. Additionally, single-center studies may have limited generalizability to other populations or settings. Therefore, our findings should be interpreted with caution, and further studies with larger sample sizes and more rigorous study designs are needed to confirm our results.

## Conclusion

In conclusion, our study suggests that a higher level of consciousness (MCS) and a negative modified Evans blue dye test (MEBDT) result may serve as potential predictors for successful decannulation in patients with prolonged disorders of consciousness (DOC). These findings highlight the importance of assessing conscious state and swallowing function in determining the feasibility of decannulation in this patient population. However, it is important to acknowledge the limitations of our study, including its retrospective nature and single-center design. Further research with larger, prospective studies is needed to validate these findings and establish more definitive conclusions regarding the predictors of decannulation in prolonged DOC patients.

## Data availability statement

The original contributions presented in the study are included in the article/supplementary material, further inquiries can be directed to the corresponding author.

## Ethics statement

The studies involving human participants were reviewed and approved by Human Ethics Committee of Huashan Hospital Fudan University. The patients/participants provided their written informed consent to participate in this study.

## Author contributions

RH introduced the blue dye test and designed this study. YC and GA imported the patient data and wrote the article. SF, TW, and LW were in charge of conducting BDT. CC, JW, and GL analyzed the data. All authors contributed to the article and approved the submitted version.

## Funding

This study was supported in part by grants from the Innovation project of Shanghai Science and Technology on Yangtze River Delta Alliance (no. 20412420200), Shanghai Municipal Key Clinical Specialty (no. shslczdzk02702), and Guiding medical project of Shanghai Science and Technology Committee (Grant no. 19411968700).

## Conflict of interest

The authors declare that the research was conducted in the absence of any commercial or financial relationships that could be construed as a potential conflict of interest.

## Publisher’s note

All claims expressed in this article are solely those of the authors and do not necessarily represent those of their affiliated organizations, or those of the publisher, the editors and the reviewers. Any product that may be evaluated in this article, or claim that may be made by its manufacturer, is not guaranteed or endorsed by the publisher.
